# Outcomes of Culturally Tailored Dietary Intervention in the North African and Bangladeshi Diabetic Patients in Italy

**DOI:** 10.3390/ijerph17238932

**Published:** 2020-12-01

**Authors:** Laura Piombo, Gianluca Nicolella, Giulia Barbarossa, Claudio Tubili, Mayme Mary Pandolfo, Miriam Castaldo, Gianfranco Costanzo, Concetta Mirisola, Andrea Cavani

**Affiliations:** 1 National Institute for Health, Migration and Poverty (INMP/NIHMP), 00153 Rome, Italy; gianluca.nicolella@inmp.it (G.N.); giulia.barbarossa@inmp.it (G.B.); miriam.castaldo@inmp.it (M.C.); gianfranco.costanzo@inmp.it (G.C.); concetta.mirisola@inmp.it (C.M.); andrea.cavani@inmp.it (A.C.); 2 Diabetes Unit, San Camillo-Forlanini Hospital, 00152 Rome, Italy; ctubili@scamilloforlanini.rm.it (C.T.); mmpandolfo@gmail.com (M.M.P.)

**Keywords:** migrants, diabetes, food habits, culturally tailored diet, transcultural mediator

## Abstract

Immigrants show higher adjusted diabetes prevalence than Italians, especially among South-East Asians followed by North and Sub-Saharan Africans. Diabetes progression is influenced by food behaviors, and diet control is a critical aspect in disease management. Food habits have many cultural and symbolic implications. Guidelines recommend that every patient should receive appropriate self-management education according to cultural and socioeconomic characteristics. This study aims to test whether a customized diet and transcultural mediator’s support can improve immigrants’ food habits. A pre-post quali-quantitative study was conducted among 20–79-year-old Bangladeshi and North African diabetic immigrants. The INMP transcultural mediator, an expert in the social and health care field, actively participates in clinical activity by decoding linguistic and cultural needs expressed by the foreigner patient. Five culturally tailored dietary profiles were designed according to international diabetes guidelines and adjusted to traditional food habits. Data were collected with two different semi-structured questionnaires. Changes in food consumption were assessed through McNemar’s test, while paired Wilcoxon Signed-Rank test was used to analyze pre and post intervention. Fifty-five patients were enrolled. At follow-up, cereals, meat, and potatoes intake significantly improved, and the number of adequate dietary habits for each patient increased significantly. Transcultural mediator support was 90% positively evaluated. Adherence to dietary control is favorably influenced by a transcultural intervention, which is based on clinical and socio-cultural criteria, in compliance with patient’s lifestyles.

## 1. Introduction

In 2019, the global international migrant population was around 272 million. International migrants in Europe were about 82 million and in Italy around 6 million, representing 10% of the whole Italian population [1]. The literature indicates a greater self-reported change in diet associated with deterioration in health with increasing length of stay [2]. Low education level, lack of employment, and negative self-perceived economic resources were conditions associated with the risk of hospitalization, longer hospital stay, and greater recourse to urgent hospitalization [3]. 

The increased migratory phenomenon created new challenges for health systems, which have to adapt to the needs of a multicultural society. World Health Organization (WHO) underlines the necessity of choosing adequate care models respectful of the patient’s culture [4]. Migrants appear more subject to communicable diseases (CDs), due to disease patterns in their origin countries, poor living conditions, precarious employment, and traumatic migratory experience [5]. However, non-communicable diseases (NCDs) are increasing among long-term resident migrants [6]. The “healthy migrant effect” tends to disappear over time. Unemployment increases and lower income also makes access to medical care more difficult, particularly among the most fragile population groups, including migrants [7]. Adult male immigrants are at higher risk of experiencing avoidable hospitalization for diabetes than Italians, with differences by area of origin, suggesting that they may experience lower access to and lower quality of primary care for diabetes [8].

Type 2 diabetes is one of the most widespread NCDs. It represents a major health burden worldwide and its incidence is increasing at an alarming rate. In Italy, immigrants show a higher adjusted diabetes prevalence than Italians [9]. Moreover, different prevalence rates exist across ethnic groups, with the highest among South-East Asians followed by North and Sub-Saharan Africans [10,11]. 

Diabetes progression is influenced by food behaviors, and diet control is one of the most critical aspects of disease management [12,13,14]. Food habits have many cultural and symbolic implications [15]. Indeed, migrants tend to maintain their traditional food habits, despite their negative impact on health. Food Balance Sheet indicate 2596 kcal/capita/day available in Bangladesh (data from FAOSTAT). The staple foods are rice and tubers and pulses (lentils) and are widely consumed. Intake of vegetables, fruit, meat, and fish is low. In North-African countries, food intake is, on average, 3300 kcal/capita/day. Staple foods are cereals (wheat, rice, corn, or barley) and pulses (chickpeas), and sugar and sweeteners are widely consumed. Intakes of vegetables, fruit (dates), and foods of animal origin is medium. The consumption of pork and alcohol is absent in all countries due to religious prohibitions.

The proposal of non-culturally tailored diets for diabetes control usually fails [16]. Therefore, strict adherence to traditional food, together with different language and religious beliefs, have been identified as an informal barrier to access to healthcare [17,18,19].

Studies have demonstrated that culturally tailored diabetes educational interventions are effective for improving glycemic control among ethnic minorities [20,21,22,23]. It was also observed that the more distant the recommended diet is from the actual habits of the patients, the more difficult it is to promote a good adherence to the nutritional therapy [24]. Guidelines recommend that every diabetic patient should receive appropriate self-management education according to specific cultural, socioeconomic, and literacy characteristics [20,25]. Cultural background influences the ability to adhere to indications for healthy eating habits among ethnic groups [26]. Nevertheless, the role of a customized diet according to immigrant culture in diabetes self-efficacy has not been highlighted in practice. The purpose of this study is to test if a culturally tailored intervention, based on customized diet and transcultural mediator’s support, can improve diabetic immigrants’ food habits.

## 2. Materials and Methods 

A pre-post quali-quantitative study was conducted at the National Institute for Health, Migration, and Poverty (INMP-Italy) outpatient clinic. The typical target of INMP are Italian and migrant disadvantaged people, refugees, asylum seekers, homeless people, victims of prostitution trade, unaccompanied minors, women with female genital mutilations, and victims of torture. Patients usually are unemployed or have casual and part-time work. Their level of education is medium-low. They often are undocumented, and cannot be registered within the Italian National Health Service. Between January 2017 and April 2018, 20–79-year-old first-generation migrants from Bangladesh, Maghreb (Tunisia, Morocco, Algeria) and Egypt were opportunistically recruited and screened for diabetes. Screening was offered to every adult patient accessing INMP outpatient clinic for an internal medicine examination and coming from the above-mentioned geographical areas. Only diabetic migrants were enrolled after written informed consent. The study was conducted in accordance with the Declaration of Helsinki, and the protocol was approved by the Ethics Committee of Ethics Committee of Istituto Superiore di Sanità (Italian higher institute for Health—PRE 565/16). Exclusion criteria included normoglycemic patients, not neo-diagnosed ones, people regularly followed elsewhere, or who declined to participate to the study. 

Two different semi-structured questionnaires were designed by the nutritionist. The baseline questionnaire, with 25 items (7 open), aimed at investigating socio-demographic characteristics, food habits, food choice determinants, barriers in dietary management, and contained a simplified food frequency questionnaire (FFQ) [27,28]. The follow-up questionnaire included the FFQ and additional 16 items (7 open) aimed at analyzing customer satisfaction, adherence to diet and the recommended activity levels. Customer’s satisfaction for perceived health conditions and the multidisciplinary care received, adherence to the diet (referred), recommended level of physical activity (referred), and usefulness of transcultural mediation were evaluated using a Likert scale ranging from (1) ‘very low’ to (5) ‘very high’.

Anthropological support was critical for including socio-cultural and migration-focused items. In particular, the anthropologist contributed to design the baseline questionnaire aimed at identifying the salient stages of the migratory path and related main breaking points that may have affected eating habits. The anthropologist’s support was also important to contextualize the social environment of the patients and highlight potential barriers to diet adherence (e.g., domestic overcrowding, loss of family ties, social hierarchy and roles, different meanings of the reasons for migration). This information was critical for the nutritionist to conduct an in-depth interview. The questionnaires were previously tested on a number of pre-diabetic patients who helped to circumscribe the list of customized questions, then included in the final questionnaire.

The multidisciplinary team included a nutritionist, a diabetologist, a medical anthropologist, medical dietitians, nurses, and three transcultural mediators (two Bangladeshi and one Arabic). The INMP transcultural mediator is an expert in the social and healthcare field who actively participates in routine clinic activity by decoding linguistic and cultural codes in the relationship between the foreign patient and Italian health professionals. In this project, transcultural mediators were also skilled on diabetes management to best contribute to a culturally oriented care model for foreign diabetic patients. Five culturally tailored dietary profiles were developed by the nutritionist supported by the diabetologist. Each one followed the international diabetes guidelines and was adjusted according to traditional food habits, as reported by the literature [29]. Based on a conventional diet for diabetic patients, profiles were designed according to traditional dishes, typical spices, and cooking techniques, giving preference to food preparations suitable for diabetic patients. In particular, the main cereals chosen for Bangladeshi and Egyptian diet were rice and wheat or derivatives such as *ruti* for Bangladesh or bread for Egypt. The main cereal used for Maghreb countries was wheat or derivatives such as bread, couscous, or pasta. For each profile, alternative menus were provided. Transcultural mediators contributed to creating a more suitable dietary profile suggesting national dishes and food, respectful of religious restrictions. Table 1 shows an extract of 2 out of 5 profiles created (only main meals/not during Ramadan). 

**Figure 1:**
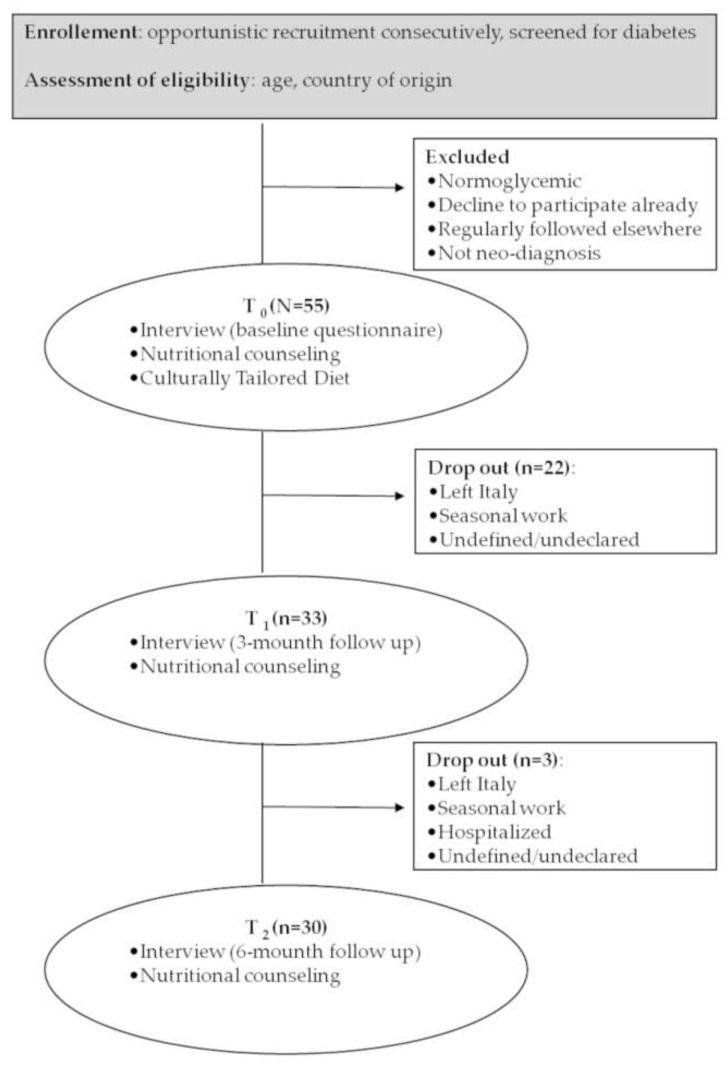
Workflow of the transcultural intervention design.

A diet plan was prepared by the nutritionist according to the patient’s culture of origin as described above. At baseline (T_0_), patients were recruited by the diabetologist according to their age and country of origin. Diabetic patients were enrolled and interviewed by the nutritionist. Information collected with baseline questionnaire (pre-questionnaire) allowed to customize each dietary profile according to patients’ lifestyle and social determinants of health, such as purchasing power, ease of consumption, and family habits. Together with the culturally tailored dietary, patients also received nutritional counseling using translated iconographic material (seasonal fruit and vegetable calendar). Patients who completed the baseline questionnaire were sent to the San Camillo-Forlanini Hospital Diabetic Center of Rome, where the dietitian finally planned a balanced intake diet. Customized diets were available both in Arabic or Bangladeshi and Italian. The administration of the 6-month-follow-up questionnaire (post- questionnaire) completed the intervention. Transcultural mediators actively participated to each setting. Figure 1 presents the study workflow.

The questionnaires were administered by the nutritionist supported by the transcultural mediator, at the baseline, and at the follow-up (after 3 and 6 months). Each T_0_ interview took about 45–50 min and each T_1-2_ one 30–40 min in a comfortable environment. Information collected with FFQ was used to classify dietary habits.

Level of food consumption was dichotomously categorized (‘adequate’ or ‘non adequate’) according to diabetes guidelines [30]. Changes in food consumption were assessed through McNemar’s test, while paired Wilcoxon Signed-Rank test was used to analyze pre- and post-intervention differences in food habits of each patient. Only differences with *p* < 0.05 were considered statistically significant.

Statistical analyses were conducted in R software (version 3.5.1; R Foundation for Statistical Computing, Vienna, Austria) [31]. Post-hoc power analysis was done by using G*Power (version 3.1.9.7; Universitat, Kiel, Germany) [32]. The incisiveness of the culturally tailored intervention was assessed using a constant comparative method (CCM).

## 3. Results

Fifty-five patients were enrolled. Table 2 describes patients’ socio-demographic characteristics, while Table 3 presents principal food choices and main barriers encountered in dietary management. Most participants were males, Bangladeshi, and living in rented accommodation with family or compatriots. Many of the interviewed were satisfied with their food habits and the majority reported to follow a typical or like-typical diet from the origin country, according to ‘their taste’ or because of ‘no real free choice’, especially those who did not live alone, or for scarce personal finances. Generic inadequate food intake and excessive sugar consumption were identified as the worst dietary habits. 

Thirty patients completed the follow up. Four out of the 25 dropouts were caused by returning to their origin country, three patients had a seasonal work far from the outpatient clinic, and one was hospitalized, whereas we do not have information about the remaining 17 dropouts. No particular socio-demographic differences were found between this group and the rest of the patients.

Patients’ food habits improved at six-month follow-up. As shown in Table 4, the number of patients with adequate consumption increased in almost all food categories. Significant changes were reported for cereals and derivatives (*p* = 0.04), meat (*p* = 0.02) and potatoes (*p* = 0.03). Furthermore, the number per patient of food categories in which adequate consumption was reached in adequate dietary habits significantly improved (*p* < 0.001). The power of Wilcoxon Signed-Rank test (1-β) turned out to be as high as 0.99 at the effect size of 1.25, α = 0.05, and the given sample size. For changes in food consumption assessed through McNemar’s test, when the significance level was set at 0.05, post hoc power analysis results of cereals and derivates, meat, and potatoes were 0.60, 0.94, and 0.77, respectively.

With regard to patients’ feedback, 93% reported a positive life change after nutrition recommendations. Customer satisfaction was generally high, both regarding perceived health conditions (average score = 3.7) and the multidisciplinary care received (average score = 4.9). Compliance to diet (average score = 3.3) and to recommended level of physical activity (average score = 3.9) was moderately good. Support from transcultural mediators was positively evaluated by 90% with an average score of 4.6. The main comments from the patients were: ‘*To better understand what my illness consists of*’, ‘*In order to communicate better with my doctor*’, ‘*For clearer information on how/when to visit*’, ‘*To better understand the dietary indications or how take medicine*’, ‘*I didn’t feel alone*’, ‘*They accompanied me to the doctor*’, ‘*Finding a person from your country who explains in your language is a fact that makes you feel comfortable, you feel welcomed*’.

## 4. Discussion

The intervention had a significantly positive effect on patients’ eating habits, a critical step for the management of diabetic patients. At the six-month follow-up, improvement in dietary habits and diabetes management was achieved. Positive effects were reported for almost all considered food categories. The best results were observed for meat, potatoes, and cereals and derivatives. Initially, their intakes were really high due to cultural food habits. Excessive daily cereal intake decreased in favor of wholegrain foods. The suitable intake of fruit and vegetables increased, as well as appropriate number of daily meals. On the contrary, snacks and soft drinks consumption were still high mainly among Bangladeshis and Moroccans. They represent typical comfort foods and a risk factor for diabetes and cardiovascular disease [33], difficult to reduce because of their gratifying power. Dietary habits in affluent countries are characterized by high sugar and carbohydrate consumption and this seems to be related to high total and cardiovascular mortality [33]. The literature indicates a greater self-reported change in diet being associated with worsening health conditions at increasing immigrants’ length of stay [2,34]. 

At baseline, food habits of patients included in the study were unhealthy, and there was urgent need to implement nutritional interventions to improve their health status. The aim of this study was to assess how the culturally tailored intervention, based on a customized diet and transcultural mediator’s support, affects dietary changes. Our study confirmed that migrants maintain their country’s food habits for multiple reasons, but it underlines that it is also possible to direct the diet towards healthy paths by leveraging cultural aspects. However, in our study, we were not able to confirm a significant relationship between the ease of adapting to a healthier diet and the immigrants’ length of stay in Italy. Cohabitation with only one cooker and economic constraints play a decisive negative influence on diet. Additionally, food choices are affected by low or absent purchasing power. Above all, in low- and middle-income patients, the use of mobile phones may be useful [35], and at the same time a technological effort is needed to protect the data collected in the design process of personalized nutritional therapies [36]. Other intervention strategies for eating behavior improvement emphasize the involvement of social and health workers of the same language in the case of Arabic patients [37]. However, strong adherence to culturally oriented diet and to religious prescriptions often puts diabetes self-management in the background when patients are not correctly informed [38]. According to Bailey et al. [39], identifying food choices determinants is essential for achieving good adherence to diet and reduce dropouts. Using traditional food to design appropriate dietary profiles has been the key to overcome cultural barriers [40]. Nutritional counseling and the choice of personalized diets have been oriented towards the eating habits of the countries of origin, promoting the healthiest ones (e.g., fish and legumes for Bangladesh, unique dishes for North Africa). The limiting factors and the barriers to access to correct lifestyles have been contextualized with the specific social determinants of the person’s health: Economic availability, time to devote to buying and preparing dishes, place of consumption of main meals, and responsible of daily nutrition. Particular attention was paid to the most dangerous categories of foods for a diabetic patient, rich in starch, correcting the excess consumption of meat. Patients’ culture and lifestyle were respected. Adapting diets to people’s cultural background can help reinforce the impact of dietary interventions. In our study, the cultural aspects of the intervention were stressed. Alongside with a culturally tailored diet, transcultural mediators were central for the success of the intervention. As evidenced by the words of the interviewees, they were fundamental to establish a ‘pact with the patient’ and support the process under both linguistic and logistic aspects. Many patients have highlighted how the mediator is ‘listening’, ‘in tune with’, activating a deeper feeling. The mediator physically accompanying the patient represented a sort of ‘protective network’, providing the patient with both critical orientation to services and psychological support.

To our knowledge there are no studies investigating the efficacy of a culturally tailored diet administered with the support of a transcultural mediator. Nevertheless, there are increasing indications that personalized dietary advice is more effective than generic nutritional information provided to consumers. Advices can be personalized in different ways: Personal preferences, self-formulated goals, eating habits, health status, or DNA profile [41,42]. Interventions of personalized medicine are also designed on the basis of behavior and social determinants such as personal and family history, cultural background, and personal beliefs and preferences [43].

Our study limitations include the lack of sex balance. Migration from Bangladesh to Italy is predominantly male and low participation of women in the sample was consistent with migratory flows. Many studies highlight the inequalities in the quality of healthcare access to specialized care and inappropriateness, due to migrant status and gender [44,45,46]. Difficulties were recorded to recruit women, notwithstanding the involvement of migrant reception centers and the organization of ‘awareness days’ in aggregation places. The role of gender differences in food supply should be investigated to understand if it can influence adherence to nutritional treatment. Due to the limited number of patients engaged, it was not possible to make any comparisons between countries of origin, nor provide clear-cut conclusion on the entire migrant population in Italy. Furthermore, although a 12-month follow-up could have been useful to confirm the improvement in eating habits, a longer follow-up was not feasible, given the characteristic mobility of the population recruited in the study. A strength of this study is that in-depth interviews allowed to get useful elements to better direct the patient towards healthier food habits, forms of cooking and preparation of the recipes, in respect of their culture and lifestyle.

## 5. Conclusions

This study underlines the importance to preventively evaluate the feasibility of nutritional therapy according to patient’s lifestyle and to analyze the difficulties actually perceived by patients in accessing and continuing care [47,48]. In order to develop appropriate nutritional interventions for diabetes care, it has been critical to first manage formal barriers, such as legal restrictions and low-income lifestyle, evaluating patient’s working hours, distance from the medical center, and returns for main meals in the reception centers.

Nutritional interventions based on both clinic and socio-cultural criteria and on the support of transcultural mediator are functional for improving diabetic immigrant’s compliance to the therapy and healthier food habits.

## Figures and Tables

**Table 1:** Culturally tailored dietary profiles for North African and Bangladeshi diabetic patients: Two main meals extract.

Main Meal	Bangladesh	Egypt
Breakfast	Salty Tea/coffee (sugar free) with *ruti* and boiled *shak* vegetable (no peas or potatoes)	Sweety Light yogurt and sugar free tea with biscuits
Lunch	A meal of parboiled/brown rice and salad as aperitif Rice with *dhal* or Rice with meat/fish curry or Rice with *shobji* and eggs or Rice with *shak*	A meal of parboiled/brown rice *Koshari* and vegetables or *Sayydaiah* and rice with vegetables or *Melokhia* with meat and rice
Dinner	*Ruti* with *shobji* (no potatoes) and light yogurt or Fish *rui*/*tengra*/*rupchada* with turmeric and *ruti* or ½ *ruti* with *aloo* or *Ruti* with *dhal*	Couscous with chickpeas/fish/meat and vegetables or Pasta with vegetables/meat/fish sauce and salad

*ruti*: Flat-bread made with millet flour, barley, wheat (without yeast); *shak*: Broad leafy vegetables (beet, spinach, chicory); *shobji*: Vegetables (zucchini, tomatoes, cauliflower, cabbage, onion, green beans, pepper, pumpkin); *salad*: Salad of lettuce, tomatoes, cucumber, onion with coriander; *dhal*: Lentils (cream); *rui*: River fish (*Labeorohita*); *tengra*: River fish (*Mystustengara*); *rupchada*: Sea fish (*Pampuschinensis*); *aloo*: Potatoes; *koshari*: A rice with pulses dish (lentils or chickpeas); *sayydaiah*: A fish dish; *melokhia*: A spinach soup.

**Table 2:** Socio-demographic characteristics of patients (*n* = 55).

Characteristic	*M* (Range)	*n*	%
Sex (n, %)			
Male		49	89
Female		6	11
Age, y (*M*, range)	44.3 (22–65)		
≤29		3	5
30-39		14	25
40–49		20	36
≥50		18	34
Origin (n, %)			
Bangladesh		37	67
Morocco		10	18
Algeria		2	4
Tunisia		1	2
Egypt		5	9
Religion (n, %)			
Muslim		53	96
Hinduism		2	4
Qualification (n, %)			
Not schooled		7	13
Junior high school diploma		28	51
High school diploma		11	20
Degree and beyond		9	16
Area of origin (n, %)			
Rural area		21	56
Urban area		24	44
Living in Italy, y (*M*, range)	11.4 (0–41)		
Accommodation (n, %)			
House rent with family or compatriots		36	66
As guest		9	16
Migrants helter		5	9
Homeless		4	7
Own house		1	2
Juridical status (n, %)			
Irregular		26	47
Regular		29	53
Work (n, %)			
Unemployed		16	29
Employed		39	71

**Table 3:** Food habits, barriers, and motivations in dietary management of patients (*N* = 55).

Items	Categories	*n*	%
Degree of satisfaction with the diet ^1^	1	6	11
	2	4	7
	3	17	31
	4	12	22
	5	16	29
How much diet have changed after arriving in Italy ^2^	1	9	16
	2	9	16
	3	14	26
	4	10	18
	5	13	24
Kind of diet followed in Italy	Only or mainly typical of own country	40	73
	Only or mainly Italian	15	27
Principal reasons	«*I prefer it*»	31	56
	«*I have no choice*»	20	36
	«*People who cook for me prefer it*»	2	4
	«*It’s cheaper*»	1	2
	«*Other*»	1	2
Most common bad dietary habits	«*I don’t know*»	32	57
	«*I eat badly (qualitatively)*»	11	20
	«*I eat too many sweets*»	7	13
	«*I often eat out of meals*»	2	4
	«*I eat too much*»	1	2
	«*Other*»	2	4
What the patient eats depends on...	«*My personal taste*»	27	49
	«*What I find ready to eat*»	13	24
	«*Taste of people I live with*»	11	20
	«*How much money I can spend*»	3	5
	«*What is given to me (food parcels, gifts)*»	1	2
Principal difficulties in management of food behaviors	«*Eating well is expensive*»	11	20
	«*I don’t have any difficulties*»	21	38
	«*I have no choice*»	7	13
	«*Laziness or lack of time*»	4	7
	«*I always have stomachache*»	2	4
	«*Respect for religious prescriptions*»	1	2
	«*Fear of forgetting the origins*»	1	2
	«*Other*»	8	14

^1^ from 1 ‘very bad’ to 5 ‘excellent’. ^2^ from 1 ‘not at all’ to 5 ‘completely’.

**Table 4:** Food frequency before and after six months (*n* = 30).

Food Categories	Patients with Adequate Consumption *n* (%)		
	Before	After	Δ
**Daily**			
Meals (4–5)	7 (23.3)	13 (43.3)	+6 (+20.0)
Fruit and vegetable (3–5)	5 (16.7)	10 (33.3)	+5 (+16.6)
Cereals and derivatives (3)	7 (23.3)	14 (46.7)	+7 (+23.4) ^1^
Milk and yogurt (1–2)	15 (50.0)	18 (60.0)	+3 (+10.0)
**Weekly**			
Meat (2–3)	3 (10.0)	12 (40.0)	+9 (+30.0) ^1^
Legumes (≥ 2)	24 (80.0)	26 (86.7)	+2 (+6.7)
Fish (≥ 2)	26 (86.7)	27 (90.0)	+1 (+3.3)
Potatoes (≤ 2)	19 (63.3)	27 (90.0)	+8 (+26.7) ^1^
Cheese (≤ 2)	24 (80.0)	27 (90.0)	+3 (+10.0)
**Monthly (rare)**			
Snack	22 (73.3)	21 (70.0)	−1 (−3.3)
Alcohol drinks	30 (100)	29 (96.7)	−1 (−3.3)
Soft drinks	16 (53.3)	18 (60.0)	+2 (+6.7)
Nr of adequate dietary habits per patient (median)	7	8	+1 ^2^

^1^ Statistically significant changes assessed by one-tail McNemar’s test *p* < 0.05. ^2^ Statistically significant changes assessed by Wilcoxon Signed-Rank test *p* < 0.05.
